# An ovarian bioreactor for in vitro culture of the whole bovine ovary: a preliminary report

**DOI:** 10.1186/s13048-016-0249-4

**Published:** 2016-08-04

**Authors:** Matthew R. Zanotelli, Joseph D. Henningsen, Patrick M. Hopkins, Aaron P. Dederich, Tessa Herman, Tracy J. Puccinelli, Sana M. Salih

**Affiliations:** 1 Department of Biomedical Engineering, University of Wisconsin-Madison, Madison, WI USA; 2 Department of Obstetrics and Gynecology, West Virginia University, Morgantown, WV 26506 USA

**Keywords:** Ovary, Ovary culture, Bovine ovary, Bioreactor, Ovarian insufficiency, Infertility, Doxorubicin, Chemotherapy, Apoptosis, Granulosa cell

## Abstract

**Background:**

Improved cancer therapeutics and enhanced cancer survivorship have emphasized the severe long-term side effects of chemotherapy. Specifically, studies have linked many chemotherapy agents with primary ovarian insufficiency, although an exact insult model has not yet been determined. To investigate and ultimately solve this problem, a novel device for extended study of mammalian ovaries in vitro was developed.

**Methods:**

A bioreactor was fabricated for bovine ovarian culture that provides intravascular delivery of media to the ovary through isolation and cannulation of a main ovarian artery branch. Whole ovaries were cultured in vitro using three methods: (1) continuously supplied fresh culture media, (2) recirculated culture media, or (3) continuously supplied fresh culture media supplemented with 500 nM doxorubicin for 24 or 48 h. TUNEL assay was used to assess apoptotic cell percentages in the three groups as compared to uncultured baseline ovaries.

**Results:**

The ovary culture method was shown to maintain cell viability by effectively delivering nutrient-enriched pH-balanced media at a constant flow rate. Lower apoptosis observed in ovaries cultured in continuously supplied fresh culture media illustrates that this culture device and method are the first to sustain whole bovine ovary viability for 48 h. Meanwhile, the increase in the percentage of cell apoptosis with doxorubicin treatment indicates that the device can provide an alternative model for testing chemotherapy and chemoprotection treatments to prevent primary ovarian insufficiency in cancer patients.

**Conclusions:**

An ovarian bioreactor with consistent culture media flow through an ovarian vasculature-assisted approach maintains short-term whole bovine ovary viability.

**Electronic supplementary material:**

The online version of this article (doi:10.1186/s13048-016-0249-4) contains supplementary material, which is available to authorized users.

## Background

While innovations in cancer chemotherapy allow patients to live longer, they also have been shown to lead to subsequent long-term complications, such as primary ovarian insufficiency (POI) [1]. POI has a pronounced effect on younger patients in particular, and can result in failure of pubertal development and complete sterility [2]. Further complications have also been associated with POI, including onset of osteoporosis and cardiovascular disease [2, 3]. These factors provide motivation to determine the cellular cause of chemotherapy-induced POI and develop drug-based measures to inhibit its development in cancer patients.

Doxorubicin (DXR) is a chemotherapy drug used to treat premenopausal cancer patients in combination with other chemotherapy protocols and has been associated with POI [4]. The effects of DXR have been documented in mice as well as marmoset ovaries including DNA damage within 2 h, follicular attrition in as little as 12 h post-injection, and permanent reduction in ovary size up to 50 % [5–7]. While embryo and oocyte cryopreservation are used to preserve fertility in reproductively-mature women [8], current methods of prevention of DXR and other chemotherapy-induced ovarian toxicity in young female cancer survivors, however, remain challenging. Methods of fertility preservation in female children and young adolescents include ovarian cortical tissue cryopreservation, an invasive and an experimental procedure [9–11]. Fertility preservation in children is in its developmental stages due to insufficient understanding of the mechanism of ovarian insult [12]. Whole ovary cryopreservation, in vitro follicle maturation, and ovarian chemoprotective drugs are also still investigational [13–22]. Further research into the mechanisms of ovarian insult could lead to more effective methods of prevention of POI during chemotherapies [20, 21].

The mechanisms behind DXR toxicity remain poorly understood. One tool to identify and study mechanisms of ovarian insult is organ culture. Most model systems for organ culture utilize bioreactors, as bioreactors have the unique ability to sustain large-scale cell viability as well as enable tissue-specific experimentation. Organ bioreactors have been successfully developed, though not yet for ovaries. Systems for isolating lungs, livers, and kidneys of large animals are commercially available and have been shown to be highly beneficial in a research setting [23–27]. In a successful organ culture, cells must be supplied with sufficient nutrition to proliferate. This is achieved with the flow of culture medium into/around the cells or tissue. Ideally, a bioreactor will meticulously control and monitor parameters that affect cell culture efficiency, namely: pH, temperature, flow rate, mechanical stresses, and biochemical gradients, to achieve an optimal culture environment. Whole ovaries have been preserved for up to 48 h under conditions that do not replicate the in vivo environment, but a whole ovary bioreactor has not been studied [28–30].

Herein, we report the design and development of a custom bioreactor for the extended culture of bovine ovaries for 48 h. This timeline was chosen to corroborate our previous mice studies where acute DXR-induced follicle apoptosis occurring within 48 h correlates with decreased fertility and reduced litter size and number throughout the reproductive lifespan of the mice [20]. Utilizing the existing ovarian vasculature, culture media was administered throughout the ovaries, and the supply of nutrients was coupled with an incubator to mimic the in vivo environment. Altogether, the system enables administration of nutrients to maintain ovarian viability, enables the introduction of DXR into the ovary to effectively study the mechanism by which ovarian insult occurs, and allows validation of putative chemoprotective drugs.

## Methods

### Ovary tissue acquisition

Bovine ovaries were obtained from the local abattoir through Applied Reproductive Technologies (Madison, WI) and transported at room temperature immediately to the laboratory. Three ovaries with pronounced vasculature were selected for each experiment. Selected ovaries were stored in 1X warmed phosphate buffered saline (PBS) (Mediatech, Inc.) until use. The University of Wisconsin - Madison approved all proceeding procedure prior to implementation.

### Bioreactor fabrication and assembly

**Fig. 1:**
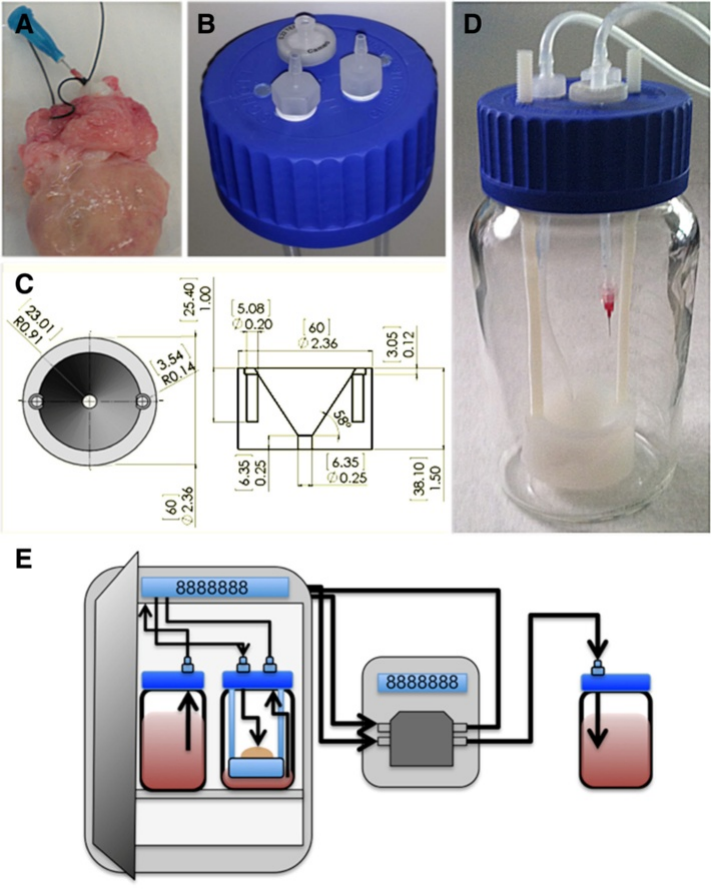
Ovarian bioreactor components and schematic of bioreactor setup. **a** Ovaries were first cannulated and tied off using suture. Ovaries were then placed in a customized bioreactor consisting of (**b**) a modified GLS 80 cap fitted with inlet and outlet ports for media flow and (**c**) a polypropylene ovary holder. **d** All bioreactor components were housed in a 1000 mL bottle with short rods connecting the ovary holder to the cap. **e** The entire bioreactor and media reservoir bottle were placed in an incubator to maintain an in vivo environment while the waste media bottle was placed outside the incubator. A digital multi-channel pump controlled and maintained media flow into the ovary via the cannula

To create a sterile, controllable environment to promote ovary maturation, a bioreactor was designed. Figure 1 demonstrates the whole ovary culture system. The ovary bioreactor was constructed from a 1000 mL Duran GLS 80 wide-mouth laboratory bottle (Sigma-Aldrich) (Fig. 1d) with a customized cap to accommodate integration with a pump. Two 1/4″ ports were drilled and tapped to provide inlet and outlet ports. Within the ports, the top of the bottle cap was fitted with 1/4″ polypropylene barbed hose adapters with 1/8″ female national pipe threads (NPT) threads (US Plastics, Lima, OH). The underside of the cap was equipped with 1/4″ polypropylene barbed hose adapters with 1/8″ female NPT threads (US Plastics) (Fig. 1b). Three 1/8″ holes were also drilled and tapped. Two of these holes were used as support for the ovary holder described below, and the third was to be fitted with a 0.22 μm air filter (Millipore) to allow diffusion of oxygen and carbon dioxide. Two additional bottles were customized in a similar fashion to serve as a fresh media reservoir and a waste media reservoir. For the reservoir bottles, each bottle cap had a single 1/4″ port drilled, tapped, and fitted with 1/4″ polypropylene threaded adapters and 1/8″ female NPT threads (US Plastics). Adapters were then connected to an 8-channel, ten rollers multi-channel peristaltic pump (Langer Instruments, model BT100-1 L) by 2250 1/8″ x 3/16″ laboratory tubing (Tygon) (Fig. 1e). The inflow loop of the system created flow from the fresh media reservoir to the bioreactor while the outflow circuit of the system removed perfused media from the ovary in the bioreactor to the waste media reservoir.

To provide support to the ovary inside the bioreactor and collect waste media, a customized polypropylene container was created (Fig. 1c). This container will be referred to as the ovary holder. To construct the ovary holder, a polypropylene sheet (MSC Direct) was milled using a Tormach p770 CNC mill (Fig. 1c). Using the CNC mill, the sheet was cut into a 2.36″ diameter cylinder that has a height of 1.50.″ From one face of the cylinder, a cone shape was removed from the center of the face to create a funnel. The cone removed from the cylinder was cut at 58° to the horizontal with a base diameter of 0.91″ and height of 1.25.″ This created the funnel which could comfortably hold a bovine ovary which is typically bean-shaped and roughly 2.0 cm x 1.5 cm. A 0.25″ diameter hole was drilled into the tip of the funnel to allow for any perfused fluid to flow out of the ovary and then exit the funnel. On the outside edge of the holder, two 1/8″ holes were drilled and tapped. Nylon rods measuring 180 mm were securely fitted and attached to the corresponding 1/8″ holes in the cap to suspend the ovary holder in the bottle. This allowed the ovary to be partially submerged in media during culture. To guide the outflow tube to the waste media located in the bottom of the bioreactor, an additional 1/4″ hole was drilled and tapped through the outer lip of the ovary holder.

### Ovary cannulation and preparation

All ovaries received were used for perfusion for the initial flow rates studies independent of whether the ovary was in the follicular or luteal phase while only ovaries in the follicular phase were used for the terminal deoxynucleotidyl transferase dUTP nick end labeling (TUNEL) assay. The procedure used for cannulation was modeled from the techniques used for perfusion of bovine and ovine ovaries in previous studies [12, 24]. The bovine ovarian artery branches higher in the mesovarium and typically 2-3 arterial branches were identified at the ovarian stump. Using sterile scissors, all extraneous ovarian tissue surrounding the vasculature was removed, leaving an approximately 5 cm stump with the ovarian vasculature remaining. A prominent ovarian artery was located within the compact nesting of vessels in the ovarian stump. The *arteria ovarica* in the vascular pedicle was identified and prepared for perfusion (Fig. 1a). The artery was not completely skeletonized as this was associated with arterial damage. The opening of the isolated artery was secured, and a 23-gauge leur stub (Instech Laboratories, Inc.) was inserted into the isolated ovarian artery and tied off using a 6-0, Prolene suture (Ethicon, US) to provide a sealed connection and prevent backflow (Fig. 1a). The leur stub was then connected via a luer adapter (Instech Laboratories, Inc.) to 1/8″ tubing (Saint-Gobain Performance PLST), which was connected to the cap and, in series, the multi-channel peristaltic pump.

Prior to experimentation, all tubing was first primed by pumping 1X PBS (Mediatech, Inc.) through the tubing and bioreactor until all air bubbles were eliminated from the tubing and cap connections. The pump was calibrated at a pressure that created a flow of 1.5 mL/min through the tubing into atmospheric conditions. Sterile 1X PBS was then pumped at this setting through the ovarian arterial vasculature for three, 5 min washes. Cannulated and washed ovaries were immediately used for testing.

### Assessment of bioreactor functionality

As the experiment was to be performed with a custom setup, the effectiveness of the bioreactor in delivering fluid to the ovary was assessed. As a proof of concept, water stained with trypan blue dye was placed in the media reservoir and run through the cannulated ovarian artery (see above). Measurements of ovary mass, fluid inflow, and fluid outflow were recorded every two min for 20 min of testing. After 10 and 60 min of the experiment, an ovary was sectioned to observe qualitatively the distribution of fluid throughout the vasculature and ovary. The complete distribution of dyed water through the vasculature and interstitial space of the ovary would indicate that the system was distributing fluid to the entire ovary over time and provide motivation for proceeding to the assessment of the setup during extended culture of whole bovine ovaries.

### Insulin-Transferrin-Selenium (ITS)

#### In vitro whole bovine ovary culture

To culture whole bovine ovaries with the system described above, ovaries were placed on the specialized polypropylene ovary holder with the leur stub oriented vertically and housed in the bioreactor. The bioreactor and fresh media reservoir were filled with 150 mL and 1000 mL, respectively, of Dulbecco’s modified Eagle’s medium/Ham’s F-12 (HyClone) supplemented with 5 % fetal bovine serum (Invitrogen), Insulin-Transferrin-Selenium (Corning), and 5 U/mL penicillin/5 μg/mL streptomycin (Gibco) [23]. The bioreactor and media reservoirs were placed in an incubator to maintain a 39 °C, 5 % CO_2_ environment while the pump and waste media reservoirs were placed next the incubator. Utilizing the chamber thru-wall access port of the incubator, tubing for all components of the system was connected (Fig. 1). Ovaries were cultured in three conditions: (1) continuous fresh media circulation (Group 1), (2) recirculation of used media (Group 2), and (3) DXR treatment of continuous fresh media circulation (Group 3). To achieve continuous fresh media circulation (Group 1), media was pumped from the fresh media reservoir and circulated through the ovarian arterial vasculature. Over time, media slowly perfused throughout the entire ovary and collected in the bottom of the bioreactor. Waste media was transported from the bottom of the bioreactor to the waste reservoir and discarded. A total of three reservoirs including the ovary bioreactor were used. Recirculation of used media (Group 2) was achieved by using two reservoirs only, and recirculating media located at the base of the bioreactor. Any media perfused from the ovary was collected and recirculated through the ovarian arterial vasculature. In the last experimental group, DXR treatment (Group 3), ovaries were cultured using the same methods as Group 1; however, culture was additionally supplemented with 500 nM DXR. All ovaries were partially submerged in 150 mL of media in the ovary holder. In all experimental groups, the driving pressure created by the pump was maintained at 1.5 mL/min under atmospheric conditions and each ovary was allowed to grow over for 2 days at 39 °C, 5 % CO_2_.

### Assessment of bioreactor environment

At 6, 12, and 24 h the fluid volume in both the fresh and waste media reservoirs was recorded. The difference in fluid volume over time in the media reservoirs was used to determine the average flow rate of the media over that period using equation 1.

1 $$ Q=\frac{\Delta V}{\Delta T} $$

Additionally, after 12, 24, and 48 h of culture, perfused media from the ovary was collected from the waste media reservoir, and pH measurements were taken to assess the culture environment created by the ovary bioreactor.

### Assessment of apoptotic cells in ovarian tissue

Immunohistochemistry was used to evaluate cellular apoptosis of ovarian tissue in the bioreactor system. Ovaries were collected after 24 to 96 h of culture and each ovary was divided into four quarters. Ovarian quarters were either fixed directly in formalin or sliced further with the Stadie Riggs Tissue Slicer to collect the superficial most layer of the ovarian cortex and then ovarian tissue was fixed in 10 % buffered formalin (Fisher Chemical) at 4 °C for ~24 h. Samples were then transferred to 70 % ethanol and stored at 4 °C until paraffin embedding and mounting. Using a microtome, samples were sliced into 5 μm sections, and a minimum of three to ten representative sections per each ovary were collected at least 25 μm intervals. Samples were stained using the ApopTag Plus Fluorescein *In Situ* Apoptosis Detection Kit (EMD Millipore) [6] with slight modifications to the manufacturer protocol [26]. Briefly, an additional pretreatment wash with 0.5 % Triton X-100 for 10 min was performed prior to applying equilibration buffer and an additional six washes with Triton X-100 were performed prior to mounting the slide. Cells undergoing apoptosis were stained with fluorescein isothiocyanate (FITC, green). All the nuclei were counterstained with 0.5 μg/mL propidium iodide (PI, red). Samples were imaged using a Nikon TI Eclipse fluorescent microscope with a motorized stage exciting/collecting at 488/525 nm for FITC and 561/570-620 for PI. Spectral images acquired utilized the spectral scan head on the A1 confocal, exciting at 488 nm and collecting emissions from 520 nm through 620 nm at 10 nm intervals [6].

Images were analyzed using Fiji (ImageJ software version 1.47v) to calculate tunnel staining in the whole ovarian sections. Images were converted to 8-bit binary using the “Process–Binary–Make Binary” option. Then utilizing the “Analyze–Analyze Particles” option (size μm^2^: 20-Infinity, circularity: 0.00-1.00) individual feature profiles of cells were counted within each image. This allowed for the number of apoptotic cells and number of total cells to be determined and used to calculate the percent of total apoptotic cells. Additionally, manual counting was used to assess TUNEL staining in the ovary as well. Manual counting allowed the careful inspection to identify ovarian structures and exclude artifacts from FITC-positive staining of red blood cells and non-nuclear staining. For manual quantification of apoptosis in ovarian stroma and granulosa cells, grids with100-μm^2^ squares were overlaid on the ovarian image. TUNEL-positive ovarian cells per randomly selected units of six squares (representing 600-μm^2^ areas) were counted [31].

### Statistical analysis

All experiments were carried out in three replicates with three ovaries for each experiment (one ovary per treatment type). All results are given as mean ± standard error. Analysis of significance was performed using a two-way ANOVA with Bonferroni tests for means comparisons (mean ± SEM) with results considered statistically significant if *p* < 0.05. Data were normalized to control to allow pooling of experiments.

## Results

### Bioreactor system setup and functionality

**Fig. 2:**
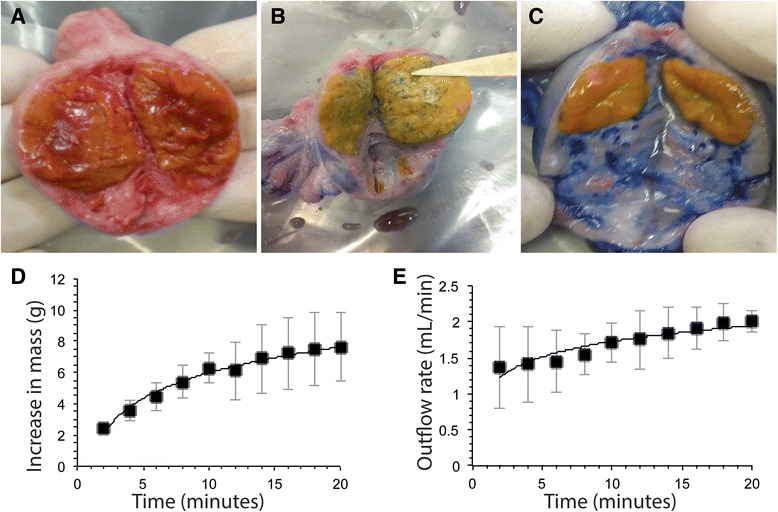
Nutrient distribution across bovine ovaries. Stained water was run through the ovarian vasculature using our cannulation method at 1.5 mL/min, and ovaries were dissected (**a**) immediately following harvest, (**b**) 10 min of fluid flow testing, and (**c**) 60 min of fluid flow testing. After extended flow, the ovarian vasculature and tissue became stained with trypan, demonstrating fluid was successfully administered via the native vasculature. **c** Over short-term culture, whole ovary mass was observed to quickly increase, but began to slow at approximately 15 min, indicating saturation (Logarithmic trendline, *R* ^2^ = 0.982), **e** Over short-term testing, a small increase in outflow rate was observed as the ovary rapidly absorbed fluid. Outflow rate subsequently began to level off as the tissue became saturated with fluid (Logarithmic trendline, *R* ^2^ = 0.885). Data presented as mean ± SE (*n* = 2)

To study the bioreactor system functionality and efficacy, we examined fluid distribution through the ovarian tissue over time. Qualitative comparison of the distribution of water dyed with trypan blue after 10 min and 60 min to uncultured control ovary demonstrated media distribution across the entire ovary via the arterial vasculature (Fig. 2a). These ovaries were not immersed in culture media to exclude diffusion of the dye from the external medium to the ovary. After 10 min of continuous flow, the ovarian vasculature and interstitial space showed signs of trypan blue staining, and after 60 min, fluid stained much of the innermost ovarian tissue (Fig. 2a-c). Importantly, the ovarian arterial vasculature remained intact in all ovaries tested. Variation in dye distribution within the ovary was also observed, as the density of the dye stained corpora lutea varies between samples. This preliminary testing demonstrated fluid distribution to the ovary via the ovarian vasculature and warranted the progression to using culture media in the system in an attempt to sustain ovary viability.

We next characterized mass changes and outflow rates from the ovary to determine if tested ovaries were absorbing administered nutrients. The average baseline weight of bovine ovaries was 15.7 ± 2.2 g. Fluid flow into the ovary was accompanied by a large increase in mass, with the ovary increasing 6.28 ± 1.9 g in mass, compared to 0 min, after 10 min. This accounts for a 40 % increase in mass. Over the following 10 min; however, the rate of mass increase slowed dramatically and only demonstrated a 7.63 ± 3.1 g increase in mass by 20 min. This accounts for a 48 % total increase, but only a 6 % increase after 10 min (Fig. 2d). The flow of dye out of the ovary exhibited a similar trend with an initially lower outflow rate of 0.933 ± 0.22 mL/min at 2 min, followed by an increased outflow rate of 1.83 ± 0.22 mL/min at 10 min, an ~97 % increase. Outflow also slowed, increasing only to over the next 10 min with a 2.01 ± 0.21 mL/min outflow rate at 20 min (Fig. 2e).

### Ovary bioreactor culture environment

To assess the bioreactor environment, we examined flow rate through the ovarian vasculature and pH of media. During the whole ovary culture, the flow rate through the ovarian vasculature of Group 1 was measured to be constant at 0.86 ± 0.02 mL/min across all time points (6, 12 and 24 h) (Table 1). The flow rate of DXR-treated samples (Group 3) were also measured to be constant with a mean of 0.88 ± 0.04 mL/min across time points and was not significantly different from the ovaries in Group 1 (Table 1). Flow rates for samples cultured in recirculated media (Group 2) could not be measured in the closed system. The pH readings of samples were varied across experimental groups. The pH of waste media from ovaries cultured with continuous fresh media circulation (Group 1) remained consistent during culture with a mean of 7.72 ± 0.04 across all time points (12, 24, and 48 h) (Table 1). Similarly, the pH of the waste media for Group 3 also remained constant 7.76 ± 0.14 across all time points (12, 24, and 48 h), similar to Group 1 (Table 1). Samples cultured in recirculated media (Group 2) showed a sharp decrease in pH, falling at each observed time point and a mean value of 6.27 ± 0.51 across the 12, 24, and 48 h time points (Table 1). Variation in both flow rate into the ovary and pH of the media over time could have an effect on cell viability during culture.

**Table 1:** Flow rate and pH measurements of the media

	Culture condition	Mean ± SEM
Average Media Flow Rate (mL/min)	Fresh media circulation (Group 1)	0.856 ± 0.023
	DXR treatment in fresh media (Group 3)	0.880 ± 0.046
Average Media pH	Fresh media circulation (Group 1)	7.72 ± 0.04
	Recirculated media (Group 2)	*6.27 ± 0.51
	DXR treatment in fresh media (Group 3)	7.76 ± 0.14

Flow rates into the ovary were calculated from fresh media circulation (Group 1) and 500 nM DXR treatments (Group 3) conditions. Flow rates are reported as bulk averages taken over 6, 12 and 24 h time points to calculate the mean and SEM at all time points. PH was measured from ovaries perfused with fresh media (Group 1), recirculation of used media (Group 2), and DXR treatment of fresh media (Group 3). Media was removed from the waste reservoir at 12, 24, and 48 h time points to measure pH and determine the effect of the different treatment groups on the bioreactor culture environment. Data presented as the mean and SE across all time points

*p < 0.05, two-way ANOVA

### Cellular apoptosis during in vitro whole bovine culture with and without DXR

**Fig. 3:**
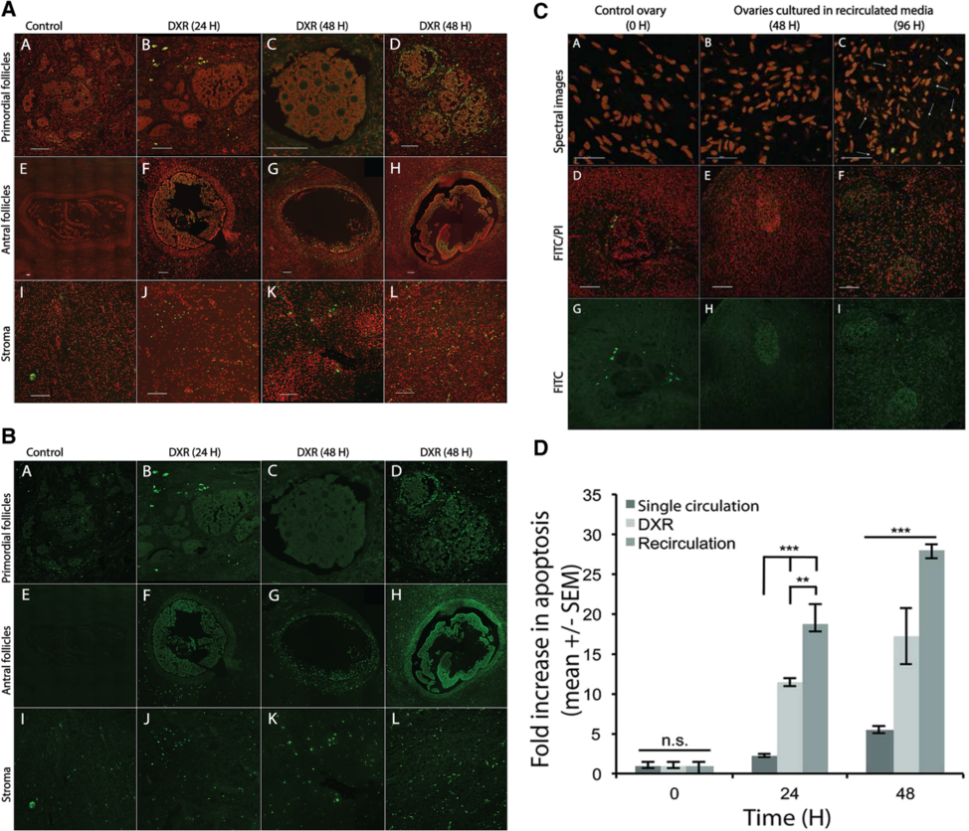
Analysis of cellular apoptosis after extended bovine ovary. **a** Representative confocal images of TUNEL staining in cultured whole bovine ovaries treated with continuous fresh media with or without 500 nM DXR for 24 h and 48 h (Groups 1 and 3). **b** represent corresponding FITC staining of the images shown in A. All treatment groups were fixed, stained using a TUNEL stain. TUNEL stain indicates cells that are undergoing apoptosis (FITC, green) and Propidium iodide (PI, red) to show the nuclei. Examples of primordial and primary follicles are shown in images A-D, antral follicles (F-H), and stroma (I to L). Control = ovaries cultured in continuous fresh media circulation, DXR = Doxorubicin. H = Hour, scale bars = 100 μm for all images. Brightness was increased to +30 to enhance image visualization. **c** Representative images of TUNEL staining of cultured whole bovine ovaries perfused with recirculated media at 0, 48, and 96 h (Group 2). A-C represents spectral composite confocal images taken at 100X oil objective and D-I represent confocal images taken at 20X objective. All treatment groups were stained using TUNEL assay. Control = uncultured ovaries. H = Hour, scale bars = 10 μm for the spectral composite confocal images (A-C). and 100 μm for the confocal images (D-I). Brightness was increased to +30 to enhance image visualization. **d** Summary graph comparing cellular apoptosis after extended culture of bovine ovaries perfused with continuous fresh media with or without 500 nM DXR (A/B), or with recirculated media (C). All treatment groups were fixed, stained using a TUNEL stain and analyzed at 24 h and 48 h time points post-DXR treatment. TUNEL-positivity was quantified in ovarian stroma and granulosa cells irrespective of follicle types. Single circulation fresh media (Group 1) demonstrated the lowest level of cell apoptosis. DXR = Doxorubicin, H = Hour; *n* = 3 ovaries per treatment group, two-way ANOVA, ** *p* < 0.01; *** *p* < 0.001, n.s = non-significant

The viability of ovaries over the course of study was measured using apoptotic markers. Specifically, ovaries in the follicular stage with no visible corpora lutea were used to assess apoptosis. Analysis of whole bovine determined that upon harvest and receipt of the ovaries, the baseline apoptotic cell percentage in fresh uncultured ovaries was 2.74 ± 2.41 % (data not shown). TUNEL. TUNEL assay results of the cultured ovaries exhibited an increase in apoptotic cell percentages from baseline as time progressed for all groups tested. The increase in apoptotic cells was, however, significantly reduced in ovaries cultured in continuously supplied fresh media (Group 1) compared to uncultured ovaries. The apoptotic cell percentage in the control ovaries cultured in continuously supplied fresh media (Group 1) was 2.2 ± 0.15 folds at 24 h and only increased to 5.4 ± 0.9 folds after 48 h, which was not different from uncultured ovaries (Fig 3a/b and d). By comparison, the increase in apoptotic cell percentage was greatest for the ovaries cultured in recirculated culture media (Group 2) as these samples exhibited an 18.8 ± 2.4 folds (*p* < 0.001) higher apoptotic cell percentage at 24 h when compared to control ovaries (Fig. 3d). This trend was consistent at 48 h, as the apoptotic cell percentage in the ovaries cultured in Group 2 increased to 27.9 ± 0.8 folds (*p* < 0.001) compared to control ovaries (Fig. 3c and d), and exhibiting FITC/PI double stained cytoplasmic granules at 96 h (arrows, Fig. 3c). This is likely due to the decrease in the pH of the recirculated culture media as noted in Table 1. Due to increase TUNEL-positive staining in this culture model, further ovarian culture experiments were performed using freshly circulated culture media (groups 1 and 3). The continuous fresh media circulation generated relatively low cell apoptosis during culture and could, therefore, be used to determine if DXR treatment affected cellular apoptosis. Ovaries cultured with DXR treatment in continuous fresh media (Group 3) exhibited an increase in apoptotic cell percentage from 11.4 ± 0.5 folds (*p* < 0.001) at 24 h to 17.2 ± 3.4 folds (*p* < 0.001) at 48 h when compared to control ovaries (Fig. 3A/B and D). At both time points, the ovaries in the DXR treatment group (Group 3) demonstrated greater apoptotic cell percentages than ovaries that were similarly cultured in continuous fresh media (Group 1), but less than that of ovaries cultured in recirculated media in Group 2 (Fig. 3d). Additional file 1: Figure S1 represents exemplar H&E images of ovaries treated with DXR at 24 and 48 h.

## Discussion

Currently, there is a paucity of in vitro models to test the toxicity of chemotherapy drugs in the ovary. In this study, we investigated the potential of a customized bioreactor to achieve extended in vitro whole bovine ovary culture. The cow offers a useful model for human ovarian disease due to the similarity of hormonal regulation of the ovarian cycle [32]. Additionally, bovine ovaries are readily available from slaughterhouses for minimal cost as they are typically discarded. In vitro fertilization procedures using oocytes harvested from bovine ovaries have been perfected and used to study morphological and molecular attributes of bovine embryo quality [33, 34]. This makes the bovine ovary a suitable model for chemotherapy toxicity testing.

Great strides have been made through in vitro culture of ovarian cells and tissue pieces/slices [32, 35, 36]. These culture methods have long been a very useful tool for studying reproductive functioning and toxicology. The highly controlled conditions that these in vitro methods provide offers insight to the mechanism of the toxicological effects of drugs such as DXR. However, many ovarian tissue cultures are not as advanced in that their preparation causes them to lose the physiological crosstalk they would have if the ovary remained whole. For this reason, new methods have been developed to better replicate an in vivo model while still maintaining a high level of control. Three-dimensional culture approaches that involve intact, 3D follicles have been developed and improved upon in mice, nonhuman primates, and humans to more accurately capture the important cell-cell communication that influences ovarian and follicular functioning [37–39]. Isolated follicle culture, however, fails to preserve the unique structural and chemical environment the entire ovary provides, in particular, the interplay between ovarian follicles and stroma. In this study, we investigated the potential of a customized bioreactor to achieve extended in vitro whole ovary culture. To the authors’ knowledge, no method for prolonged in vitro culture of mature, large animal ovaries currently exists. Whole organ culture preserves the intricate interaction between somatic and germ cells, as well as recapitulates the oocyte, follicle, and stroma interactions in a more comprehensive fashion. Additionally, whole ovary culture provides the opportunity to assess ovarian connective tissue and vasculature in their innate environment. Here, we effectively utilized a vasculature-assisted method to distribute nutrients throughout the entire ovary, providing an environment mimicking in vivo conditions while maintaining low cellular apoptosis during culture. Additionally, the ease of use and degree of control offered through our bioreactor allows for quick and straightforward environment alteration to enable use for different species. Thus, we conclude that our system creates a suitable environment for successful mature, whole bovine ovary culture as well as demonstrates the potential use for studying the ovary, particularly the effects of chemotherapy agents on the ovary.

The development of a system to efficiently deliver nutrient to the entire organ is a fundamental piece to achieving successful whole ovary culture. To achieve prolonged in vitro culture of follicles it is vital to ensure nutrients reach the innermost of the ovary, which has a rich vascular supply, to support growing follicles [3]. Other whole organ cultures, such as small mammal ovary culture, recirculate media over the course of culture [40]; however, this was observed to be ineffective in whole bovine ovary culture. When media was recirculated through the ovary a significant decrease in culture media pH and apoptotic cells was observed, indicating an extremely harsh culture environment. Here, we utilized catheterization of the *arteria ovarica* to provide nutrients throughout the ovary via the vasculature. This method of catheterization has been previously used for cryopreservation as well as for nutrient delivery to other systems including the Langendorff heart [40]. In our vascular-assisted perfusion model, only the prominent branch of the ovarian arteries at the ovarian stump was cannulated and perfused. Prior studies estimated bovine ovarian blood flow rate at 1–3.2 mL/min depending upon presence or absence of corpus luteum [41]. A flow rate of 1.5 mL/min representing the median of the reported range was used in our study.

After administering trypan blue dye to the ovary via the vasculature at a constant flow rate, the ovary demonstrated a gain in mass and outflow rate was initially lower than inflow rate. The ovarian weight increased up to 7.63 ± 3.1 g (~50 %) in the first 20 min. This is equivalent to approximately 7500 μL of the medium being held in the ovary, indicating ovarian tissue absorbed the delivered fluid prior to fluid diffusion spontaneously out of the ovary. The initial increase in ovarian mass may have occurred because fluid initially accumulated in the ovarian interstitial space prior to diffusing freely out of the ovary. Despite the increase in ovarian weight, this did not appear to increase the percentage of apoptosis when compared to uncultured ovaries. Over time, the rate of mass increase slowed, as the tissue became saturated and outflow rate nearly equaled inflow rate. These observations mimic in vivo conditions previously described [13, 16]. Flow rate results paralleled distribution of dye through the vasculature and interstitial space of the ovary, with dye staining most of the ovarian interstitium tissue after 60 min. The observed variation in the staining of the corpora lutea is likely due to the selective cannulation of the most prominent ovarian artery branch. As no rupture of the vasculature was observed and the trypan blue dye appeared to travel smoothly through the cannulated vasculature, we conclude this is an effective method to deliver nutrients.

The chemotherapy drug, DXR, has well documented negative effects, but the mechanism behind its toxicity is not well understood. In order to elucidate the toxicity mechanism, a more effective in vitro model is needed. Thus, we examined the apoptotic effects of DXR, in order to evaluate our system for chemotherapy toxicity testing. DXR treatment demonstrated significant differences in the percentage of apoptotic events seen between the DXR (Group 3) and the group using the continuous fresh media circulation (Group 1) provides preliminary validation that this method will be useful for the study of ovarian tissue. The DXR group had a substantial increase in apoptotic events showing that altering the bioreactor culture conditions changes the ovary tissue response. Adding DXR to the culture media yielded a response consistent with expectations [1, 10, 32] providing further evidence that the bioreactor provides a controllable and reliable means for media to interact with the entire ovary. Whole organs respond to external factors differently than isolated tissues. The method presented here appears to provide an efficient way of studying how the whole ovary responds to DXR while also mimicking the environment that the ovary will experience in vivo. Further testing and optimization of the chemical environment used for whole ovary culture will elucidate the effectiveness of culture technique as well as the potential for long-term whole ovary culture and will allow assessment of the biological and functional characterization of the system. Future studies will assess longer periods of in vitro ovary culture to determine the maximum duration that the ovary could maintain its viability and the best time point to assess ovarian toxicity. Since the bovine ovarian artery branches higher prior to reaching the ovarian hilum, future studies will assess the need to cannulate multiple ovarian artery branches in this model. Standardization of the age and breed of the animals and the use of ovaries from young heifers is expected to decrease tissue heterogeneity while allowing the assessment of chemotherapy-induced ovarian toxicity and the biological significance of morphological and molecular changes in the ovary in greater detail. In this study, we report apoptosis in stroma and granulosa cells without reference to particular follicle types. Although we did not quantify apoptosis per follicle type due to the heterogeneity of the tissue and lack of a sufficient number of different follicle types in some sections, general apoptosis throughout the ovary was significantly increased post-DXR treatment indicating the validity of the model. Future studies will aim at a modified technique that includes serial sectioning of larger portions of the bovine ovary in younger cows to capture adequate numbers of various follicle types. This will allow assessment of apoptosis per follicle type while avoiding the repeated counting of preantral and antral follicles which has an estimated diameter of 100 – 200 μM and 15–18 mm, respectively [42].

## Conclusions

In summary, an effective method of culturing large animal ovaries was developed and tested. The ovary bioreactor system was shown to maintain low levels of cellular apoptosis for at least 48 h of culture and provided the ovary with necessary nutrients without inflicting rupture of the perfused ovarian vasculature. Methods described here not only provide a method for large, mature ovary culture and study of follicular development but also provide a method for drug screening. Observing the effects of drugs, particularly chemotherapy drugs, on the whole ovary will provide insights that are not offered in traditional in vitro follicle cultures.

## Abbreviations

DXR, Doxorubicin; FITC, Fluorescein isothiocyanate; NPT, National pipe threads; PBS, Phosphate buffered saline; PI, Propidium iodide; Q = ΔV/ΔT, Q indicates flow rate (mL/min); ΔV indicates change in volume (mL); ΔT indicates change in time (min); SEM, Standard error of the mean; TUNEL, Terminal deoxynucleotidyl transferase dUTP nick end labeling

## Additional file

Additional file 1: Figure S1. Representative H&E staining of control and DXR-treated whole bovine ovaries. Panels **A-D** display primordial follicles, while panels **E-H** display antral follicles. Images **E-H** are an exact replicate of the corresponding images in Fig. 3. Brightness was increased to +30 to enhance image visualization. (TIF 43 mb)
