# Supplementary figures and images for: An ovarian bioreactor for in vitro culture of the whole bovine ovary: a preliminary report
Source: J Ovarian Res. 2016 Aug 4;9:47. doi: 10.1186/s13048-016-0249-4 (PMC4973044; doi:10.1186/s13048-016-0249-4)

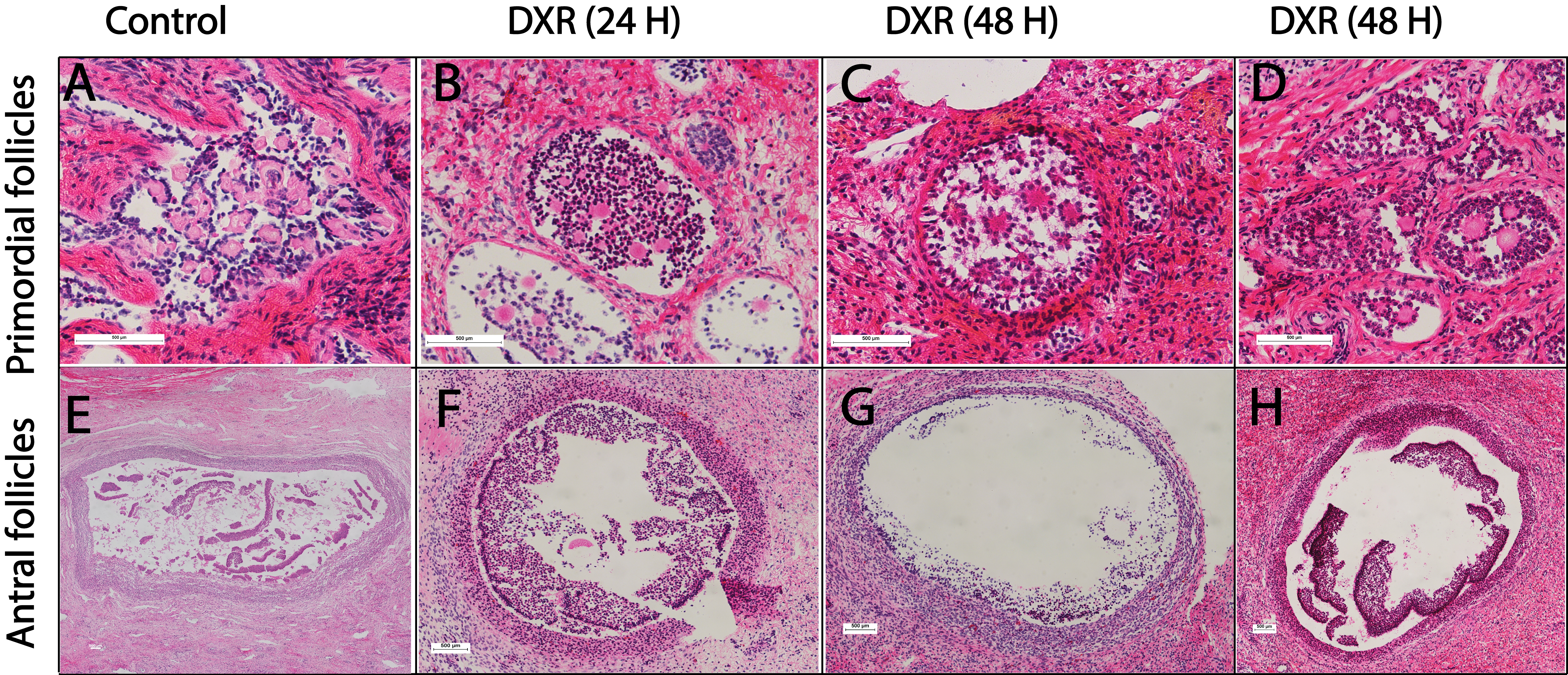

Supplement: Additional file 1: Figure S1. — Representative H&E staining of control and DXR-treated whole bovine ovaries. Panels A-D display primordial follicles, while panels E-H display antral follicles. Images E-H are an exact replicate of the corresponding images in Fig. 3. Brightness was increased to +30 to enhance image visualization. (TIF 43 mb) [file 13048_2016_249_MOESM1_ESM.tif]
